# Exploring Microdiversity in Novel *Kordia* sp. (Bacteroidetes) with Proteorhodopsin from the Tropical Indian Ocean via Single Amplified Genomes

**DOI:** 10.3389/fmicb.2017.01317

**Published:** 2017-07-25

**Authors:** Marta Royo-Llonch, Isabel Ferrera, Francisco M. Cornejo-Castillo, Pablo Sánchez, Guillem Salazar, Ramunas Stepanauskas, José M. González, Michael E. Sieracki, Sabrina Speich, Lars Stemmann, Carlos Pedrós-Alió, Silvia G. Acinas

**Affiliations:** ^1^Department of Marine Biology and Oceanography, Institut de Ciències del Mar, Consejo Superior de Investigaciones Científicas Barcelona, Spain; ^2^Bigelow Laboratory for Ocean Sciences, East Boothbay ME, United States; ^3^Department of Microbiology, University of La Laguna La Laguna, Spain; ^4^National Science Foundation, Arlington VA, United States; ^5^École Normale Supérieure, Département de Géosciences, Laboratoire de Météorologie Dynamique, UMR 8539 ENS-CNRS- École Polytechnique Paris, France; ^6^Sorbonne Universités, UPMC Université Paris 06, CNRS, Laboratoire d’Océanographie de Villefranche (LOV) UMR7093, Observatoire Océanologique Villefranche-sur-Mer, France; ^7^Systems Biology Program, Centro Nacional de Biotecnologia, Consejo Superior de Investigaciones Científicas Madrid, Spain; ^8^Departament de Genética i de Microbiologia, Facultat de Biociències, Universitat Autònoma de Barcelona Bellaterra, Spain

**Keywords:** MLSA, SAGs, Bacteroidetes, *Kordia*, proteorhodopsin

## Abstract

Marine Bacteroidetes constitute a very abundant bacterioplankton group in the oceans that plays a key role in recycling particulate organic matter and includes several photoheterotrophic members containing proteorhodopsin. Relatively few marine Bacteroidetes species have been described and, moreover, they correspond to cultured isolates, which in most cases do not represent the actual abundant or ecologically relevant microorganisms in the natural environment. In this study, we explored the microdiversity of 98 Single Amplified Genomes (SAGs) retrieved from the surface waters of the underexplored North Indian Ocean, whose most closely related isolate is *Kordia algicida* OT-1. Using Multi Locus Sequencing Analysis (MLSA) we found no microdiversity in the tested conserved phylogenetic markers (16S rRNA and 23S rRNA genes), the fast-evolving Internal Transcribed Spacer and the functional markers proteorhodopsin and the beta-subunit of RNA polymerase. Furthermore, we carried out a Fragment Recruitment Analysis (FRA) with marine metagenomes to learn about the distribution and dynamics of this microorganism in different locations, depths and size fractions. This analysis indicated that this taxon belongs to the rare biosphere, showing its highest abundance after upwelling-induced phytoplankton blooms and sinking to the deep ocean with large organic matter particles. This uncultured *Kordia* lineage likely represents a novel *Kordia* species (*Kordia* sp. CFSAG39SUR) that contains the proteorhodopsin gene and has a widespread spatial and vertical distribution. The combination of SAGs and MLSA makes a valuable approach to infer putative ecological roles of uncultured abundant microorganisms.

## Introduction

The phylum Bacteroidetes is the third most abundant group of bacteria in the oceans (Kirchman, 2002) but has been poorly studied at the species level compared to the two other main marine microbial phyla, i.e., Proteobacteria and Cyanobacteria. Bacteroidetes is a cosmopolitan phylum that typically constitutes between 4 and 22% of marine bacterioplankton cells (Glöckner et al., 1999; Cottrell and Kirchman, 2000; Alonso-Sáez et al., 2007; Ruiz-González et al., 2012; Lefort and Gasol, 2013; Acinas et al., 2014). The relative abundance of Bacteroidetes can reach up to 53% and it often correlates with phytoplankton blooms (Abell and Bowman, 2005; Fandino et al., 2005). Bacteroidetes are proficient at the degradation of particulate organic matter (POM) (Cottrell and Kirchman, 2000; Gómez-Pereira et al., 2012; Fernández-Gómez et al., 2013; Swan et al., 2013) and present a generally high growth rate when substrate is available (e.g., following phytoplankton blooms) (Kirchman, 2002; Ferrera et al., 2011; Buchan et al., 2014). Some representatives contain the gene coding for the light-dependent proton-pump proteorhodopsin, that has been shown to stimulate bacterial growth or work as a source of additional energy in the presence of light in some strains (Gómez-Consarnau et al., 2007; Stepanauskas and Sieracki, 2007; González et al., 2008; Woyke et al., 2009; Martínez-García et al., 2012). Although Bacteroidetes can be found in the free-living plankton size fraction, they seem to be predominantly particle-attached (DeLong et al., 1993; Schattenhofer et al., 2009; Crespo et al., 2013; Díez-Vives et al., 2014; Salazar et al., 2015).

Population genetics studies of marine Bacteroidetes are scarce, and have been generally conducted using very small populations of isolates or comparing several distant species (Fernández-Gómez et al., 2013; Swan et al., 2013), giving a general overview of common genotypic traits and differences above the species level. Single cell sequencing enables the retrieval of discrete microbial genomes from natural samples. A direct link between phylogenetic markers and functional information from single cells allows a finer resolution in microdiversity studies (Stepanauskas and Sieracki, 2007). When combined with multi locus sequencing analysis (MLSA), this approach can easily provide a robust phylogenetic reconstruction of the selected population. MLSA has been widely used for population genetics analyses since it is a feasible approach for delineating ecologically relevant units within species (Gevers et al., 2005). It is based on the sequencing of several marker genes and so far, its use in marine bacterial taxa has been limited to a few cultured groups such as *Alteromonas macleodii* (Ivars-Martínez et al., 2008), members of *Vibrio* (Thompson et al., 2005) including *Vibrio cholerae* (Boucher et al., 2011), *Tenacibaculum maritimum* (Habib et al., 2014), or *Prochlorococcus* (Kashtan et al., 2014). Since it is well known that many ecologically relevant bacterial taxa elude isolation in culture (Rappé and Giovannoni, 2003), the combination of single cell genomics of environmental samples and MLSA can help in the exploration of intra-specific genetic variability as well as the different evolutionary processes involved in microbial speciation (such as homologous recombination and divergent selection).

The genus *Kordia* (Bacteroidetes, Flavobacteriaceae) contains representatives with isolation sources suggesting different lifestyles and habitats: (i) marine surface seawater for *Kordia antarctica* (Baek et al., 2013), *K. aquimaris* (Hameed et al., 2013), and *K. algicida* (Sohn et al., 2004), (ii) surface freshwater for *K. zhangzhouensis* (Lai et al., 2015), (iii) the interphase between the ocean and a freshwater spring for *K. jejudonensis* (Park et al., 2014), (iv) the surface of the green alga *Ulva* sp. for *K. ulvae* (Qi et al., 2016) or (v) the digestive tract of a marine polychaete for *K. periserrulae* (Choi et al., 2011).

Here we report the first MLSA analysis of the genus *Kordia*, performed with 98 Single Amplified Genomes (SAGs) from the genus *Kordia* that were retrieved from a seawater sample from the North Indian Ocean, a location subjected to seasonal monsoon winds and coastal upwelling events as well as open ocean oligotrophy. As a result, the microbial community structure varies and large phytoplankton blooms are common, especially those formed by diatoms (Landry et al., 1998). One characteristic feature of the North Indian Ocean is the marked Oxygen Minimum Zone (OMZ) layered in the subsurface of the upwelling regions (Roullier et al., 2014). Even though there have been studies of the bacterial community composition of the Indian Ocean seawater (Sunagawa et al., 2015; Wang et al., 2016) and deep-sea sediments (Wu et al., 2011; Khandeparker et al., 2014), the diversity and genomics of the Bacteroidetes populations remain poorly characterized in this area. This is then a good opportunity to analyze whether micro-diversity exists in a particular Bacteroidetes taxon dominating after the phytoplankton bloom event. We used the well-conserved ribosomal RNA genes, the fast-evolving internal transcribed spacer (ITS) and the beta subunit DNA-directed RNA polymerase *rpo*B. We also screened these genomes for a gene that can provide ecological information of the targeted population: proteorhodopsin (PR), which is present in ecologically relevant Bacteroidetes genomes (González et al., 2008; Pinhassi et al., 2016). Fragment Recruitment Analysis (FRA) of metagenomic reads suggested the preferred habitat and an estimated abundance of the novel *Kordia* sp. CFSAG39SUR in different oceans, depths and size fractions.

## Materials and Methods

### Sampling, Generation and Selection of Single Amplified Genomes

**FIGURE 1:**
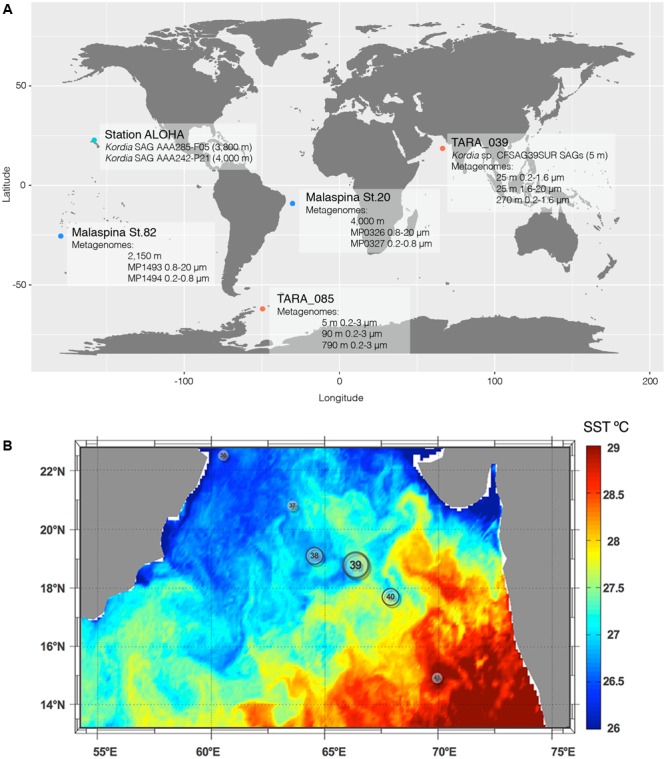
Global map showing the location of all relevant SAGs and metagenomic samples used in this study **(A)** and Sea Surface Temperature of *Tara* Oceans stations visited in the north Indian Ocean leg **(B)**. Station TARA_039 was where the *Kordia* SAGs where retrieved from and is located at a mesoscale front between the warm waters of the Indian Ocean basin and the cooler waters from the coastal upwelling.

Surface (SUR) seawater samples (5 m deep, not pre-filtered, Sample ID: TARA_G000000266) were collected for single cell analysis from the North Indian Ocean during the circumnavigation expedition *Tara* Oceans, station TARA_039 ^1^ (**Figure 1**). The environmental setting of the station was inferred from its physical report and can be found at Pangaea website in the following link: http://store.pangaea.de/Projects/TARA-OCEANS/Station_Reports/TARA_039_oceanographic_context_report.pdf.

Replicated 1 mL aliquots of seawater were cryopreserved with 6% glycine betaine (Sigma) and stored at -80°C. Samples used in this study were collected from TARA_039 (SUR; 5 m deep) located in the North Indian Ocean (18.59–66.62) on March 18th, 2010 (Supplementary Table 1). SAGs generation and preliminary 16S rRNA screening with primers 27F and 907R (Supplementary Table 2) were performed at the Bigelow Laboratory Single Cell Genomics ^2^. More information can be found in Swan et al. (2011). Partial 16S rRNA gene sequences (640–850 bp) were compared to previously deposited sequences using the RDP Naive Bayesian rRNA Classifier Version 2.4 tool, and 98 SAGs with >99% similarity to the reference strain *K. algicida* OT-1 (AY195836) were selected for further analyses. These represented 84% of successfully amplified SAGs (total of 117 SAGs). They were named CFSAG39SUR referring to their taxonomic assignment (Cytophaga-Flavobacteria), retrieval method (SAG) and sampling station (TARA_039, SUR). Other SAGs used in this study (AAA285-F05 and AAA242-P21) were collected at 3,000 m at 4,800 m, respectively, in the North Pacific Sub-tropical Gyre. They belong to the Hawaii Ocean Time-Series (HOT, **Figure 1A**) and were generated following the same procedures.

### Amplification of Phylogenetic Markers 16S rRNA, ITS and 23S rRNA Genes

Amplification of the 16S rRNA gene, ITS and 23S rRNA gene was done by a single Polymerase Chain Reaction (PCR) amplification with the 358F and CF434R primers (Supplementary Table 2). Amplification was performed in a Biorad Thermocycler by an initial denaturation at 94°C for 5 min, followed by 40 cycles of 94°C for 1 min, 55°C for 1 min and 72°C for 2 min, and a final extension at 72°C for 10 min. Each amplification reaction contained: 3 to 20 ng of template DNA, dNTPs (200 μM each), MgCl_2_ (2 mM), primers (0.5 μM each), Taq DNA polymerase (1.25 U), the PCR buffer supplied by the manufacturer (Invitrogen, Paisley, United Kingdom) and MilliQ water up to the final volume of 25.

### Amplification of Functional Genes: *rpo*B and Proteorhodopsin

A set of newly designed primers was used for *rpo*B amplification (Supplementary Table 2). Each PCR reaction followed an initial denaturation at 94°C for 5 min, 40 cycles of 94°C for 1 min, 40°C for 45 s and 72°C for 1 min, and a final extension at 72°C for 5 min. Each amplification reaction contained: 1.5 to 10 ng of template DNA, dNTPs (0.2 mM each), MgCl_2_ (1.5 mM), primers (0.25 μM each), KAPA2G Robust HotStart DNA Polymerase (1 U), KAPA2G Buffer A (KAPA BIOSYSTEMS, Wilmington, MA, United States) and MilliQ water up to the final volume of 25 μl. For proteorhodopsin (PR) amplification, the primers used and the PCR protocol were those described by Yoshizawa et al. (2012) (Supplementary Table 2) using the KAPA2G Robust HotStart DNA Polymerase and KAPA2G Buffer A from KAPA BIOSYSTEMS. Each PCR reaction followed an initial denaturation at 94°C for 5 min, 35 cycles of 94°C for 1 min, 44°C for 45 s and 72°C for 1 min, and a final extension at 72°C for 5 min. Each amplification reaction contained: 3 to 20 ng of template DNA, dNTPs (0.2 mM each), MgCl_2_ (3 mM), primers (0.5 μM each), Taq DNA polymerase (1.25 U), the PCR buffer supplied by the manufacturer (Invitrogen, Paisley, United Kingdom) and MilliQ water up to 25 μl.

### Sequencing and Phylogenetic Analysis

Polymerase Chain Reaction products were purified and sequenced by Genoscreen (Lille, France) with OneShot Sanger sequencing and aligned against NCBI’s nBLAST and xBLAST databases for identification. Reference sequences used in this study were retrieved from Integrated Microbial Genomes and Metagenomes (IMG) 4 Data Management or NCBI database. Using Geneious Pro 4.8.5 software all sequences were aligned (using default Geneious progressive pairwise aligner) trimmed and manually checked. The software was also used to obtain the complete 16S rRNA gene assembling the amplicon sequences from MDA screening and the phylogenetic markers amplification. Alignments were processed into phylogenetic trees with Mega 5.2.2 Software choosing Maximum Likelihood statistical methods along with 1000 bootstrap replications. The best-fit substitution models for Maximum Likelihood phylogenies of the studied markers were: GTR with Gamma distribution (16S rRNA gene), Kimura-2 with Gamma distribution (23S rRNA gene) and WAG with Gamma distribution (PR and RpoB). Representatives from the Proteobacteria and Cyanobacteria phyla were used as outgroups in all phylogenies. Phylogeny reconstruction of functional genes used the closest sequences to the SAGs’ genes obtained from the Ocean Microbiome Reference Gene Catalog (Sunagawa et al., 2015).

### Fragment Recruitment Analysis (FRA)

Nucleotide-Nucleotide BLAST 2.2.28+ was used to recruit metagenomic reads from several metagenomic samples similar to all the available sequenced *Kordia* genomes. Considering the aim of this part of the study, which was to determine the presence and distribution of the novel *Kordia* sp. CFSAG39SUR in different oceanic regions, depths and size fractions, a database containing all 4 *Kordia* genomes was generated. This ensured a competitive recruitment between the genomes for each metagenomic sample analyzed. The quality and genome completeness of the four reference genomes in the database was measured with software checkM (Parks et al., 2015) and fetchMG (Sunagawa et al., 2013) (Supplementary Table 3). The available genomic sequences of *Kordia* AAA285-F05 did not code for any of the marker genes or COGs used by the software, resulting in a genome completeness estimation of 0% despite being 283.58 Kb long. The other three reference genomes (*K. algicida, K. jejudonensis*, and *K. zhangzhouensis*) were estimated to be complete and free of contamination. Recruitment regarded only one read per target gene (-max_target_seqs 1), an identity percentage higher than 70% (-perc_identity 70), as well as an *e*-value lower than 0.000001 (-e-value 0.000001). All other nBLAST settings were set on default. In order to avoid random alignments, a filtering process was applied using R software (R Core Team, 2013) (i) excluding alignments with coverage lower than 90% of the sequence length, (ii) removing duplicated reads within each genome and (iii) masking reads belonging to the ribosomal operon. This operon does not follow the species delimitating recruitment patterns as the rest of the genome does and its presence would overestimate read recruitment (Caro-Quintero and Konstantinidis, 2012). All contigs from each genome were concatenated to a single sequence and the file containing the four sequences (one for each genome) was used to generate the database. This step was done using NCBI’s *makeblastdb* application. As query we selected those metagenomic samples which contained the most metagenomic reads 16S rRNA and metagenomic Illumina tags (miTags) (Logares et al., 2013) annotated as *Kordia* (data not shown) using SILVA database Release 115 (Quast et al., 2013). These were stations TARA_039 (0.2–20 μm) and TARA_085 (0.2–3 μm) from *Tara* Oceans (Supplementary Table 1 and **Figure 1A**) (Sunagawa et al., 2015). In addition, we used 4 metagenomes from the deep waters of Malaspina 2010 circumnavegation expedition (Acinas et al., in preparation), two of them from the Brazil Basin in the Atlantic Ocean and two of them from Circumpolar Deep Waters (Supplementary Table 1 and **Figure 1A**). Due to the flexibility of Bacteroidetes’ lifestyle, the metagenomic selection was done from a limited pool of deep ocean metagenomes covering the free-living fraction of prokaryotes (0.2–0.8 μm) and the fraction of prokaryotes attached to particles or aggregates (0.8–20 μm) of a same seawater sample. The coverage of different deep ocean biomes and the highest abundance of *Kordia* related metagenomic read recruitments was also taken into account for the metagenomes’ selection for the study. All queries consisted on merged, paired-end reads with different sequencing depths. Merging was done using Mothur v.1.33.3 (Schloss et al., 2009). Recruited data normalization was done: (i) by reference genome size (resulting in recruited reads/genomic bp), then (ii) upscaling this ratio to 1 Mb (as done in Swan et al., 2011), and finally (iii) by metagenomic sequencing depth. As sequencing depths varied between metagenomes, normalization was performed to the smallest metagenomic sequencing depth of the study.

### Accession Numbers

Accession numbers for PCR products of *Kordia* CFSAG39SUR: 16S rRNA gene (MF187452), ITS (MF187454), 23S rRNA gene (MF187453), *rpo*B (MF187456), and proteorhodopsin (MF187455). Accession numbers for partial 16S rRNA gene sequences are: MF187458 for AAA285-F05 and MF187457 for AAA242-P21.

The genomes used as reference for the FRA are the following: Bacteroidetes bacterium SCGC AAA285-F05 (JGI GoldStamp Id Go0060979), *K. algicida* strain OT-1 (NCBI RefSeq NZ_ABIB00000000.1), *K. jejudonensis* strain SSK3-3 (NCBI RefSeq NZ_LBMG00000000.1), *K. zhangzouensis* strain MCCC 1A00726 (NCBI RefSeq NZ_LBMH00000000.1). The *Tara* Oceans metagenomes used as query are the following (INSDC run accession numbers):TARA_039_DCM_0.22-1.6 (ERR599145), TARA_039_MES_0.22-1.6 (ERR599037| ERR599172), TARA_085_SRF_0.22-3 (ERR599090| ERR599176), TARA_085_DCM_0.22-3 (ERR599104| ERR599121), TARA_085_MES_0.22-3 (ERR599008| ERR599125). The Expedición Malaspina metagenomes are the following (JGI Genome Portal, genome.jgi.doe.gov/): MP1493 (/DeeseametaMP1493_FD), MP1494 (/DeeseametaMP1494_FD), MP0326 (/DeeseametaMP0326_FD), MP0327 (/DeeseametaMP0327_FD) with the previous agreement of the PI of the JGI CSP 602 grant.

## Results

### Environmental Setting and Bacterial Diversity of Station TARA_039

On February 2010, 1 month prior to sampling, a moderate bloom of phytoplankton occurred in the Oman Gulf due to a coastal upwelling under strong wind conditions. It propagated to the central basin of the Arabian Sea reaching only up to station TARA_039 (see Figure 3 in Roullier et al., 2014), which was the last mesotrophic station occupied by the *Tara* Oceans expedition before entering the warm oligotrophic waters of the central basin. Previous stations occupied during this leg in the North Indian Ocean (TARA_037 and TARA_038) were in the cooler waters of the upwelling, whereas station TARA_039 was at a mesoscale frontal region with the oligotrophic zone (**Figure 1B**). Particles produced during the phytoplankton bloom decreased in size as the cruise reached the central basin (see Figure 10 in Roullier et al., 2014). Diatom contribution to total phytoplankton decreased from 20 to 5% from stations close to the Gulf of Oman down to station TARA_40, outside the upwelling waters. Sunagawa et al. (2015) found that the bacterial assemblage at station TARA_039 was dominated by Proteobacteria in both surface and DCM (Deep Chlorophyll Maximum) layers, followed by Cyanobacteria and other, less abundant groups including Euryarchaeota, Actinobacteria, Deferribacteres, and Bacteroidetes (Sunagawa et al., 2015). In the mesopelagic (MES) zone there was a decrease in the abundance of Proteobacteria, a small increase of Deferribacteres and Actinobacteria and a remarkable increase in Thaumarchaeota and unclassified Bacteria. There was an increase in Bacteroidetes abundance toward the end of the *Tara* Oceans’ sampling leg in the North Indian Ocean (Sunagawa et al., 2015).

### Phylogenetic Reconstruction of a Natural *Kordia* Population

**FIGURE 2:**
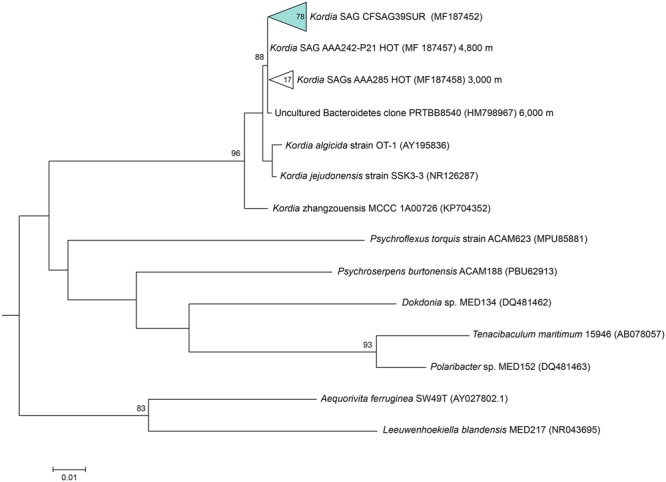
Maximum likelihood tree based on partial 16S rRNA gene sequences (805 bp), showing relationships between the representative sequence of the identical 16S rRNA gene from the *Kordia* SAGs (CFSAG39SUR) and members of the Bacteroidetes phylum. Only bootstrap values ≥ 70% are shown at the nodes. All the sequences have been retrieved from the JGI IMG database with the exception of AAA242-P21 and AAA285 that are unpublished SAG sequences.

Amplification of the three phylogenetic markers of the ribosomal operon was successful in 78 out of 98 *Kordia* SAGs, and their alignment indicated a 100% identity among them (**Table 1**). The resulting sequences contained full 16S rRNA gene (1,495 bp), ITS-1 (536 bp) and partial 23S rRNA gene (415 bp) (**Table 1**). The alignment of the resulting full 16S rRNA gene sequence against NCBI database assigned the SAGs to the genus *Kordia*, family Flavobacteriaceae. Its best hit with a nucleotide identity of 99% and sequence coverage of 97% was an uncultured Bacteroidetes sampled in the Puerto Rico Trench (clone PRTBB8540, acc. HM798967) at 6,000 m depth. The closest sequenced cultured relatives were *K. algicida* strain OT-1 with a 96.8% of nucleotide identity and 99% coverage (complete sequence, acc. NR027568) and *K. jejudonensis* strain SSK3-3 with the same identity percentage but 96% coverage (partial sequence, NR_126287). A 100% nucleotide identity was found with partial 16S rRNA gene sequences from 17 SAGs collected at 3,000 m (AAA285) and one SAG from 4,800 m depth in the North Pacific Sub-tropical Gyre (AAA242-P21), which belong to the Hawaii Ocean Time-series (HOT) (Stepanauskas, unpublished results). The phylogenetic reconstruction based on the representative sequences of the 16S rRNA genes from the 78 SAGs (**Figure 2**) strongly indicated that they belonged to the genus *Kordia* (phylum Bacteroidetes, family Flavobacteriaceae). High bootstrap values supported the cluster formed by the 78 CFSAG39 SAGs with the 17 AAA285-SAGs, the one AAA242-P21 SAG and the uncultured Bacteroidetes clone PRTBB8540. This cluster’s sequences were all retrieved from deep waters except for the SAGs reported here. The resulting sequence from the 78 identical partial 23S rRNA genes, when aligned against the NCBI database, showed a lower identity against *K. algicida* OT-1 (90.5%). Nevertheless, it was still its closest culture hit and the phylogenic tree supported the assignment of the SAGs to the genus *Kordia* ITS with high bootstrap values (Supplementary Figure 1).

**Table 1:** Summary on genetic information of *Kordia* Single Amplified Genomes (SAGs) and closest SAGs, isolates and clones. Identity % against *Kordia* sp. CFSAG39SUR’s corresponding genes (16S, ITS and 23S) and proteins (RpoB and PR).

*Kordia* sequence information	Origin	Depth (m)	16S		ITS		23S		*rpo*B		PR	
			# seq.	id %	# seq	id %	# seq	id %	# seq	id %	# seq	id %
CFSAG39SUR (98 SAGs)	North Indian Ocean	5	78	100	78	100	78	100	18	100	34	100
AAA242-P21 (1 SAG)	NPSG ALOHA	4000	1	100	–	–	–	–	–	–	–	–
AAA285 (17 SAGs)	NPSG ALOHA	3800	1	100	–	–	–	–	–	–	–	–
*Kordia algicida* OT-1 (1 isolate)	Masan Bay, S.Korea	0	3	96.8	3	76.1	3	90.5	1	89.3	0	–
*Kordia* sp. PC-4 (1 isolate)	Sagami Bay, Japan	100	1	98.4	–	–	–	–	–	–	1	89.2
Unc. Bact. PRTBB8540 (1 clone)	Puerto Rico Trench	6000	1	99.9	–	–	–	–	–	–	–	–

*For each gene and protein, “# seq” refers to the number of sequences available for comparison.*

The 78 SAGs’ ITSs were identical and coded for Ala-tRNA and Ile-tRNA. These tRNAs showed only one polymorphism each when compared to those of *K. algicida*. The nucleotidic change was located at the next-to-last base, not affecting the structure of the functional molecule. The box A conserved region present at the end of the ITS was identical to that of *K. algicida* although the former was located slightly upstream (17 bp) (Supplementary Figure 2).

Amplification of the beta subunit of RNA polymerase (*rpo*B) was successful in 18 SAGs, obtaining ∼1,400 bp amplicons with 100% of both amino acid and nucleotide identity among them. Their closest hit in NCBI database was the *rpo*B gene of *K. algicida* OT-1 with a 98% amino acid identity. High bootstrap values supported the location of the SAGs’ *rpo*B sequence in the phylogenetic tree, within the Flavobacteriaceae cluster and closest to *K. algicida* OT-1 (Supplementary Figure 3).

An amplification summary can be found in **Table 1**.

### Novel Proteorhodopsin Gene

**FIGURE 3:**
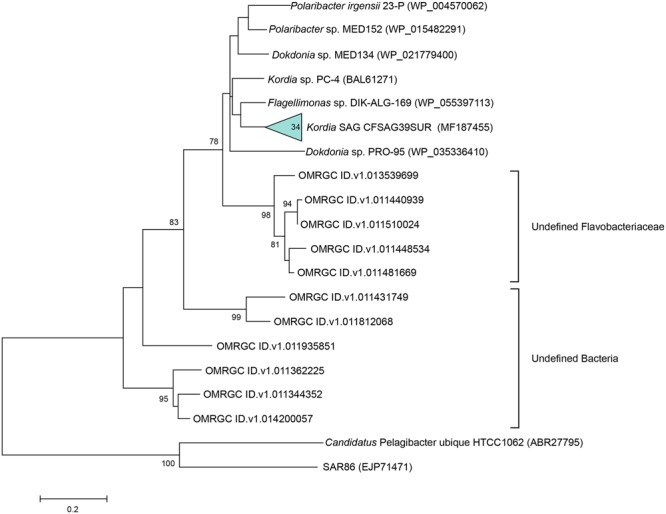
Maximum likelihood tree based on partial proteorhodopsin amino acid sequences (167 aa), showing relationships between the representative sequence of the 34 identical PR genes from the *Kordia* SAGs (CFSAG39SUR), the closest hits according to NCBI and the Ocean Microbiome Reference Gene Catalog. Other sequences were retrieved from the JGI IMG database. *Candidatus* Pelagibacter ubique and SAR86 act as outgroups. Only bootstrap values ≥ 70% are shown at the nodes.

The PR gene was amplified in 34 out of the 98 *Kordia* SAGs. The resulting 167 amino acid sequences turned out to be 100% identical to one another. The sequence was closest to the Flavobacteriaceae *Flagellimonas* sp. DIK-ALG-169’s proteorhodopsin (89% identity, 100% coverage) (AHN13811) and *Kordia* sp. PC-4 (Yoshizawa et al., 2012) (88% identity, 91% coverage) in the NCBI database. When comparing sequences in the Ocean Microbiome Reference Gene Catalog (OMRGC), the closest hit shared 84.2% amino acid similarity (unclassified Flavobacteria, NOG136807 ID.v1.022398661). Phylogenetic reconstruction placed the SAGs’ proteorhodopsin in a cluster of Flavobacteriaceae PR genes (**Figure 3**). We tried to amplify the proteorhodopsin gene from the 17 SAGs retrieved from 3,000 m at the North Pacific Gyre (AAA285-F05) but the results were negative.

**FIGURE 4:**
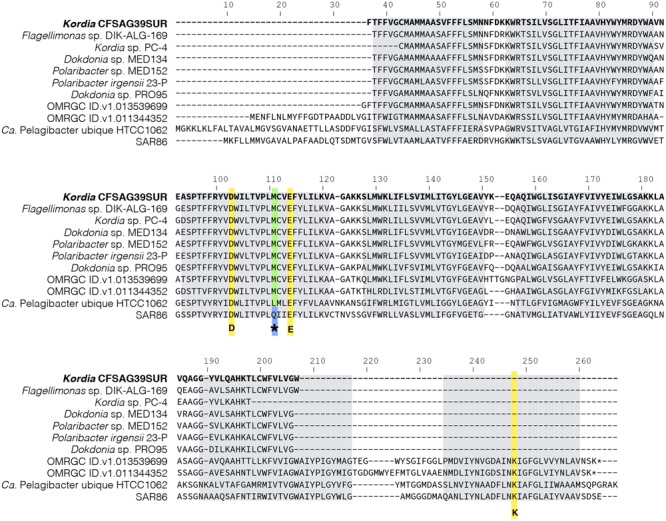
Amino acid alignment of full and partial proteorhodopsin representative sequences from Bacteroidetes (*Kordia* SAGs CFSAG39SUR sequence in bold at the top) and some outgroups. Key amino acids for proteorhodopsin functionality are highlighted in yellow: D (Asp) and E (Glu) are the proton acceptor and donor, respectively, conforming the Schiff base. K (Lys) is the amino acid to which retinal binds. The amino acid that plays a role in spectral tuning is marked with ^∗^. Methionine (M) and leucine (L) lead to absorption maximum of green light, between 518 and 535 nm whereas glutamine (Q) sets the absorption maximum of blue light, ∼490 nm. Predicted transmembrane helices are highlighted as gray boxes.

The SAGs’ proteorhodopsin alignment with other closely related PR sequences showed that this proteorhodopsin had the same structural features as its relatives. It had the predicted transmembrane helices, key amino acids for functionality and the location of the spectral tuning amino acid, which in this case was methionine (**Figure 4**), indicating absorbance of light spectrum between 518 and 535 nm (green light).

### Distribution of Novel *Kordia* sp. in Different Oceanic Regions

Metagenomic reads from different stations were recruited through a competitive FRA with the available sequenced genomes of the genus *Kordia:* the deep-sea *Kordia* SAG AAA285-F05 (283.58 Kbp), the seawater surface isolated in culture *K. algicida* OT-1 (5.01 Mbp), the freshwater *K. zhangzhouensis* MCCC 1A00726 (4.03 Mbp) and *K. jejudonensis* SSK3-3 (5.3 Mbp), isolated from a region where spring freshwater and seawater meet. We counted reads with 70–100% identity to these reference genomes.

**FIGURE 5:**
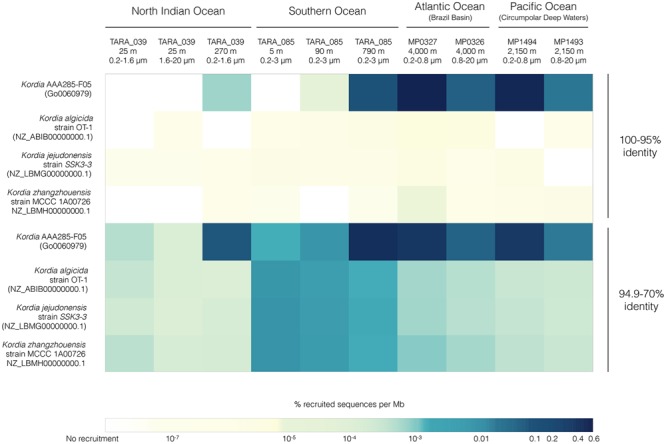
FRA results for the four available *Kordia* genomes mapped against 10 metagenomic samples from different locations, depths and size fractions. Heatmap picturing percentage of recruited reads per genomic Mb normalized by metagenomic sequencing depth and reference genome size. The upper four rows depict recruitment at 100-95% identity against reference genome, the bottom four rows depict recruitment identity values between 70 and 94.9%.

For *Kordia* AAA285-F05, the number of recruited reads increased with depth in all stations tested. The percentage of reads recruited per genomic Mbp was several orders of magnitude larger in the free-living than in the particle associated size fractions (**Figure 5**; numeric data in Supplementary Table 4).

Highest relative abundances of reads per Mbp with 95–100% identity for *Kordia* AAA285-F05’s occurred in the free-living fraction of bathypelagic waters from the Brazil Basin (MP0327, 0.51%) and the Circumpolar Deep Waters of the Pacific Ocean (MP0326, 0.46%). Recruitment percentages for this SAG in these two locations decreased down to 0.04 and 0.01%, respectively. In TARA_039 highest recruitment % per Mbp was 0.0007% at 270 m, while in the Southern Ocean (TARA_085), recruitment increased with depth reaching 0.06% at 790 m. Recruitment was virtually zero for the isolates genomes at this recruitment identity percentage.

Recruitment reads with 70–94.9% identity might be indicative of populations related to, but different from, the reference genome present at the tested metagenomic sample. The recruitment trend observed in the higher identity percentage for *Kordia* SAG AAA285-F05 was consistent at the lower percentage as well. Recruitment % per genomic Mbp increased at all depths of both TARA_039 and TARA_085, now reaching values of 0.05% at 270 m in TARA_039 and the maximal of 0.38% at 790 m in TARA_085. Similar abundances were obtained for the Brazil Basin (MP0327, 4,001 m) and the Circumpolar Deep Waters (MP1494, 2,150 m) in their free-living fraction (0.3 and 0.27%, respectively). Recruitment decreased in the larger size fraction of the two Malaspina 2010 circumnavigation expedition metagenomes down to 0.04 and 0.01%, respectively. For the other three *Kordia* genomes competing for recruitment in the same metagenomic samples, recruitment values decreased significantly to values ranging from 0.008% per Mb for *K. zhangzhouensis* in TARA_085 surface waters to values down to 10^-7^% per Mb. The recruitment for these three genomes followed a similar pattern, with highest values in TARA_085 surface decreasing with depth. In their case, bathypelagic samples and North Indian ocean’s DCM (25 m) recruited similar values (∼0.0002%). The lowest values were found at TARA_039 mesopelagic waters (0.00001%).

**FIGURE 6:**
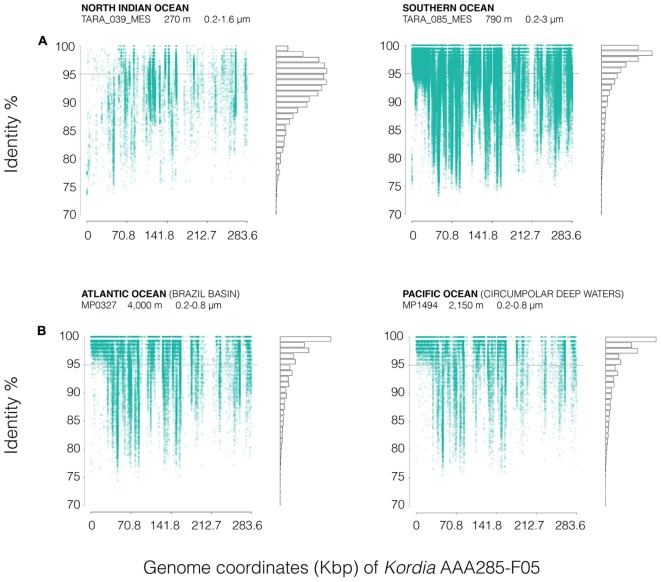
FRA plots of *Kordia* SAG AAA285-F05 partial genome (283.58 kb), against **(A)** two different stations from the *Tara* Oceans metagenomes (TARA_039_MES and TARA_085_MES) and **(B)** two metagenomes from the Malaspina 2010 circumnavigation expedition deep metagenomes (MP0326 at 4,000 m deep and MP1494 at 2,150 m deep). FRA has been done competitively between the four available *Kordia* genomes. Metagenomic reads are displayed according to their position against de reference genome (*X*-axis, genome coordinates) and their identity percentage (*Y*-axis). The gray line located at 95% identity in the *Y*-axis corresponds to 95% average nucleotide identity (ANI), threshold for bacterial species categorization. The bars on the right show the amount of reads mapped at each identity percentage. Duplicates and reads mapped to the ribosomal operon have been removed to avoid bias.

FRA plots (**Figure 6**) showed a similar pattern of *Kordia* AAA285-F05 recruitment for the meso- and bathypelagic metagenomes (TARA_085_MES, MP0327, MP1494) of the free-living prokaryotic fraction. There was a very good coverage of the whole reference sequence, with the exception of some fragments where there was a visible decrease in the recruitment at all identity percentages. The highest read densities were found above 95% identity. In the TARA_039 OMZ recruitment plot, an overall decrease in read density was observed, identities ranged from 85 to 99%. The two genomic regions with very low recruitment were also apparent in this plot.

The three reference *Kordia* genomes (Supplementary Figure 4) showed read clouds at the lower identity percentage in all cases. There was a good coverage of the length of the genome but not as intense as that observed in **Figure 6** for reference genome *Kordia* AAA285-F05.

## Discussion

The aim of this study was to explore the potential microdiversity within a population of 98 SAGs of the genus *Kordia* (*Kordia* sp. CFSAG39SUR), a Flavobacteriaceae genus established after describing *K. algicida* strain OT-1 (Sohn et al., 2004). Having these SAGs was a unique opportunity to study the extent of microdiversity within a large population of environmental uncultured organisms, since genomic length and composition of marine uncultured bacterial genomes (SAGs) generally differ from those of similar taxa isolated in pure culture (Swan et al., 2013).

Phylogenetic proximity among *Kordia* sp. CFSAG39SUR SAGs was confirmed by the 100% identity of the full 16S rRNA gene, partial 23S rRNA gene, and the ITS-1 region. The latter is located between the 16S and 23S ribosomal genes and has been widely used when more detailed taxonomical resolution was needed, especially in intra-specific diversity studies (Brown and Fuhrman, 2005). Moreover, its variability in length and composition highlights initial genome diversification and evolutionary speciation (Schloter, 2000). The 100% identity at the nucleotide level of functional genes *rpo*B and PR (exposed to a high degree of horizontal gene transfer, Fuhrman et al., 2008) could suggest that the genetic homogeneity may be present throughout the whole genome. However, only the analysis of the whole genomes would confirm it or reject this hypothesis.

Such genetic similarity among SAGs can be attributed to two main factors: (i) to stable environmental conditions providing fewer mutation-prone events, hence low microdiversity in the population (Cohan, 2006), or (ii) recent population expansion, as nascent populations are usually more genetically homogenous than mature ones, in which products of mutations and recombination may accumulate (Cordero and Polz, 2014). As mentioned above, a month prior to the arrival of the *Tara* schooner to the North Indian Ocean, a coastal upwelling fertilized the surface waters of the Gulf of Oman with new nutrients, resulting in a phytoplankton bloom that extended through the coast of Iran and south-east to station TARA_039 (Roullier et al., 2014). These blooms have been described to be seasonal, mostly formed by diatoms (Landry et al., 1998). The large particles generated by the bloom were seen to decrease in size through the sampling period (Roullier et al., 2014). Backward Lagrangian particle transport modeling suggested that these particles sunk near station TARA_039 (Roullier et al., 2014), where a frontal system separated the colder upwelled waters from the warmer waters of the oligotrophic basin. Considering the occurrence of such an event before the sampling at TARA_039, it is highly possible that the genetic homogeneity found in the 78 *Kordia* sp. CFSAG39SUR SAGs is due to the second possibility mentioned above, that is, that these bacteria were retrieved as a nascent population derived from the phytoplankton bloom.

There is previous knowledge of the relationship between members of the genus *Kordia* and algae. The first isolate described for the genus, *K. algicida* OT-1, was isolated following a red tide of the diatom *Skeletonema costatum* (Sohn et al., 2004). Additionally, it is the only Bacteroidetes species found to code for R-bodies, a specific system for targeted lysis of eukaryotes (Raymann et al., 2013). Likewise, *K. ulvae* was isolated from the surface of the marine alga *Ulva* sp. (Qi et al., 2016). Abundant algal products or exudates could provide a good environment for the rapid proliferation of our *Kordia* sp. CFSAG39SUR SAGs either attached to large particles or free-living in the water column. This would help to explain the apparent lack of microdiversity found at the time of sampling and that would probably be due to nascent populations from a single genotype after the algal bloom. Interestingly, a population of 18 SAGs identical to this *Kordia* sp. CFSAG39SUR population at the available partial 16S rRNA gene sequence were retrieved from the deep waters of station ALOHA in the North Pacific Subtropical Gyre, where upwelling and seasonal diatom blooms are common events (Villareal et al., 2012).

The *Kordia* sp. CFSAG39SUR SAGs made 84% of the sorted heterotrophic genomes retrieved from the sample, suggesting a high abundance of *Kordia* at the moment of sampling. Even though there was no metagenomic sample available from TARA_039 surface waters to support this high abundance and being aware of the environmental context of the sampling, we relied on FRA to test its persistence through the water column. Metagenomic read recruitment of *Kordia* spp. was very low in the TARA_039 DCM metagenome and slightly higher in TARA_039 MES metagenome. These values pointed to *Kordia* sp. CFSAG39SUR SAGs as members of the less abundant taxa known as the rare biosphere (Pedrós-Alió, 2012). Higher abundances (up to 0.51% of recruited reads per genomic Mbp) were found in other metagenomic samples from *Tara* Oceans and Malaspina 2010 circumnavigation expedition covering different ocean locations, depths (0–4,000 m) and size fractions (0.2–20 μm). In many cases read abundances reached values similar to recruitments in the surface waters of different oceans using as reference isolated Rickettsiales, *Planctomyces* or Rhodobacterales, generally considered to be abundant bacteria. Nevertheless, read abundances never reached those of isolated *Prochlorococcus, Synechococcus*, or *Pelagibacter*, the most abundant groups in seawater (see Supplementary Figures S10, S11 by Swan et al., 2011). The scarce recruitment in TARA_039 metagenomes contrasts with the high abundance of SAGs retrieved from the surface waters of the station. We think it is important to highlight the fact that the sampled water processed for single cell genomics was not prefiltered. Together with the slight increase in recruitment at the mesopelagic layer, we cannot reject the possibility that sequences related to *Kordia* sp. CFSAG39SUR SAGs could have been abundant in metagenomes of larger size fractions (20–200 μm) than those available (0.2–20 μm), especially since large particles (2–2.5 mm at 520–560 m, see Figure 10 in Roullier et al., 2014) were reported at Station TARA_039 deeper waters during the *Tara* expedition. It is possible then, that this new *Kordia* species becomes abundant only after upwelling-induced phytoplankton bloom events. To further test the preferred niche for *Kordia*, FRA should be performed on metagenomes of higher planktonic size fractions, either from the same stations or from stations where diatom blooms occur after upwelling episodes. Unfortunately, such metagenomes are not currently available.

This suggests a hypothesis where, after colonization of phytoplankton-derived large particles, *Kordia* sp. CFSAG39SUR would sink with the particles to the deep ocean, becoming part of the bacterial seed bank of the rare biosphere. In fact, FRA of reference genome *Kordia* SAG AAA285-F05 displayed higher read recruitments (i.e., relative read abundance per genomic Mbp) in metagenomes from deeper water samples. Intriguingly, our *Kordia* sp. CFSAG39SUR SAGs coded for a light dependent proteorhodopsin, a metabolic advantageous trait in sunlit water layers. The sequence was similar to those of other Flavobacteria which have been shown to absorb green-light, and have fast photocycles characteristic of proton-pumping rhodopsins rather than sensing rhodopsins (Spudich, 2006). This suggests that *Kordia* sp. CFSAG39SUR has an active metabolism in the upper layers of the ocean. Perhaps their activity decreases or becomes dormant when reaching bottom waters. There, they would accumulate attached to the remaining particles or switching to a free-living lifestyle, explaining the highest recruitments at the free-living fraction of meso- and bathypelagic waters. It must be considered, however, that proteorhodopsins have been found in the deep sea (Tang et al., 2015; Yoshizawa et al., 2015) and that both presence and expression of proteorhodopsin genes were detected in the Arctic Ocean during the Polar night (Nguyen et al., 2015), suggesting that proteorhodopsins may have an unknown function in dark waters.

Comparative genomics has helped us infer a species threshold based on Average Nucleotide Identity (ANI, Konstantinidis and Tiedje, 2005) where 95% identity corresponds to the previous 70% DNA–DNA hybridization threshold, which was the gold standard for taxonomic species identification (Wayne et al., 1987). FRA on metagenomes (Caro-Quintero and Konstantinidis, 2012) using a reference genome makes it possible to infer: (i) similar genetic populations (“species”) above >95% ANI and (ii) different sequence-discrete populations within the range of 80–95% ANI. For this study, as the *Kordia* sp. CFSAG39SUR genome sequences are not available, we carried out the analyses with the genome of *Kordia* SAG AAA285-F05 as reference because they shared 100% identity in their 16S rRNA genes. Despite this identity at the 16S rRNA level, there may be significant differences both at the gene content level, as well as in the identity between shared genes. It is common that within a given bacterial species there is a set of genes conserved among all strains for housekeeping functions (core genome), while an array of different functional genes can vary depending on the niche of the specific bacteria (flexible genome) (Medini et al., 2005; Fernández-Gómez et al., 2012). Despite this limitation, the fact that we used *Kordia* AAA285-F05 as our reference genome still may provide an indirect detection of *Kordia* sp. CFSAG39SUR SAGs through the FRA of the fraction of the genome shared between the reference and our SAGs. Since we do not know to what extent *Kordia* sp. CFSAG39SUR resembles *Kordia* SAG AAA285-F05 apart from the 16S rRNA gene in those shared genes, reads recruited between identity values of 95–100% would reveal the presence of *Kordia* belonging to the same species as *Kordia* AAA285-F05. If our *Kordia* sp. CFSAG39SUR on the other hand, was actually a co-occurrent relative of the reference but not the same ecotype (i.e., nucleotidic differences in those shared genes), we would indirectly detect it in those reads recruited below 95% identity (Caro-Quintero and Konstantinidis, 2012).

Being aware of this, we also used all available *Kordia* spp. genomes as reference for the FRAs at the same time. This analysis confirmed that those reads mapping against AAA285-F05’s genome at lower identity ranges belonged, indeed, to a new ecotype from the same novel species (likely our *Kordia* sp. CFSAG39SUR SAGs). The non-existent recruitment of reads at 95–100% for those genomes different from *Kordia* AAA285-F05 backs it up strongly.

## Conclusion

This study shows how state-of-the-art single cell genomics can be combined with more traditional techniques such as MLSA to obtain an overview of the genetic composition of the population of study without sequencing whole genomes beforehand. Thus, this can provide both an analysis of population genetics avoiding bias by isolation in culture and an approach to select genomes of interest for further population genomics and biogeography studies. In our case, through MLSA of SAGs we have found that the dominant heterotrophic bacterial taxon in the sample was genetically homogeneous. Moreover, the study of this population in combination with available metagenomic datasets and the oceanographic metadata of the sampling area has helped shedding light on the species distribution, dynamics, and potential ecological niche of a novel *Kordia* species that contains proteorhodopsin.

## Author Contributions

SGA designed this research. MR-L performed the laboratory and analyses work and wrote the paper. IF, FC-C, GS, and PS helped with the data analyses. SS and LS contributed to oceanographic dataset and comments of the sampling station. MS and RS contributed with the *Tara* Ocean SAGs collection and sorting. SGA funded this research with contributions from CP-A and JG. All authors were involved in critical reading for writing the paper with special contribution of CP-A.

## Conflict of Interest Statement

The authors declare that the research was conducted in the absence of any commercial or financial relationships that could be construed as a potential conflict of interest.

## Footnotes

1 oceans.taraexpeditions.org

2 scgc.bigelow.org
