# Supplementary material for: Exploring Microdiversity in Novel Kordia sp. (Bacteroidetes) with Proteorhodopsin from the Tropical Indian Ocean via Single Amplified Genomes
Source: Front Microbiol. 2017 Jul 25;8:1317. doi: 10.3389/fmicb.2017.01317 (PMC5525439; doi:10.3389/fmicb.2017.01317)
Supplement: Supplementary file 1 [file Data_Sheet_1.pdf]

Exploring microdiversity in novel *Kordia* sp. (Bacteroidetes) with proteorhodopsin from the tropical Indian Ocean via single amplified genomes

Royo-Llonch M, Ferrera I, Cornejo-Castillo FM, Sánchez P, Salazar G, Stepanauskas R, González JM, Sieracki ME, Speich S, Stemmann L, Pedrós-Alió C and Acinas SG*

Supplementary Material

Figures:

Fig 1. 23S rDNA tree
Fig 2 Box A region against *Kordia algicida'*s Box A
Fig 3. rpoB tree
Fig 4. Recruitments of Kordia spp. in TARA_039_MES

Tables:

Table 1. Stations' information
Table 2. Primers used in this study
Table 3. Genome completeness and quality estimations
Table 4. FRA values

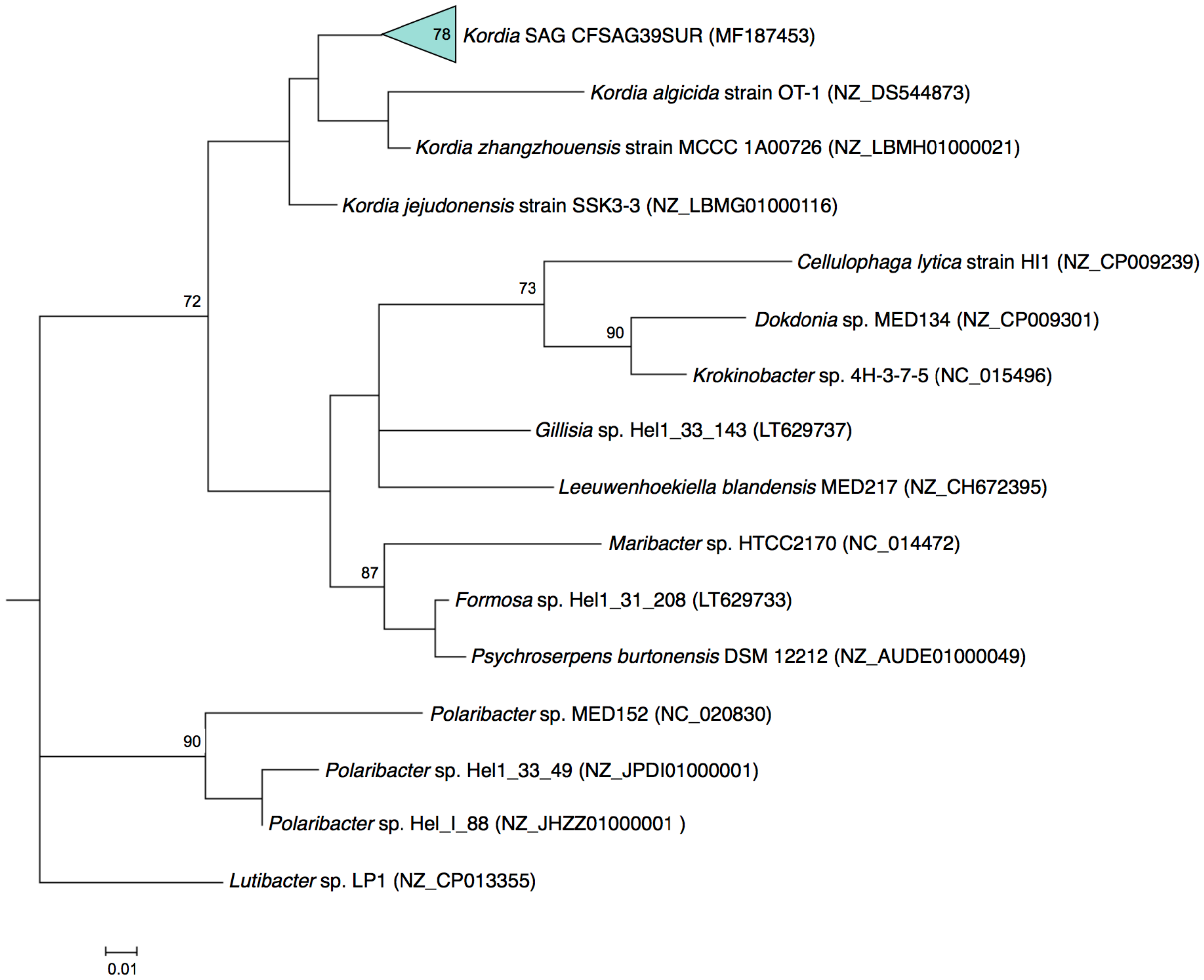

Figure 1. Maximum likelihood tree based on partial 23S rRNA gene sequences (415 bp), showing relationships between the representative sequence of the identical 23S rRNA gene from the 78 *Kordia* SAGs (CFSAG39DCM) and members of the Bacteroidetes phylum. Only bootstrap values ≥ 70% are shown at the nodes. All the sequences have been retrieved from the JGI IMG Database.

Consensus Box A for phylum Bacteroidetes		5’ – G T T C T/A T T G A C A T A T T G – 3’
Kordia SAG CFSAG39SUR	ITS bp 442	5’ – G T T C A T T G A C A T A T T G – 3’
Kordia algicida OT-1	ITS bp 459	5’ – G T T C A T T G A C A T A T T G – 3’

Figure 2. BoxA sequences of *Kordia* SAG CFSAG39SUR and *Kordia algicida* OT-1 aligned to consensus Box A sequence for phylum Bacteroidetes.

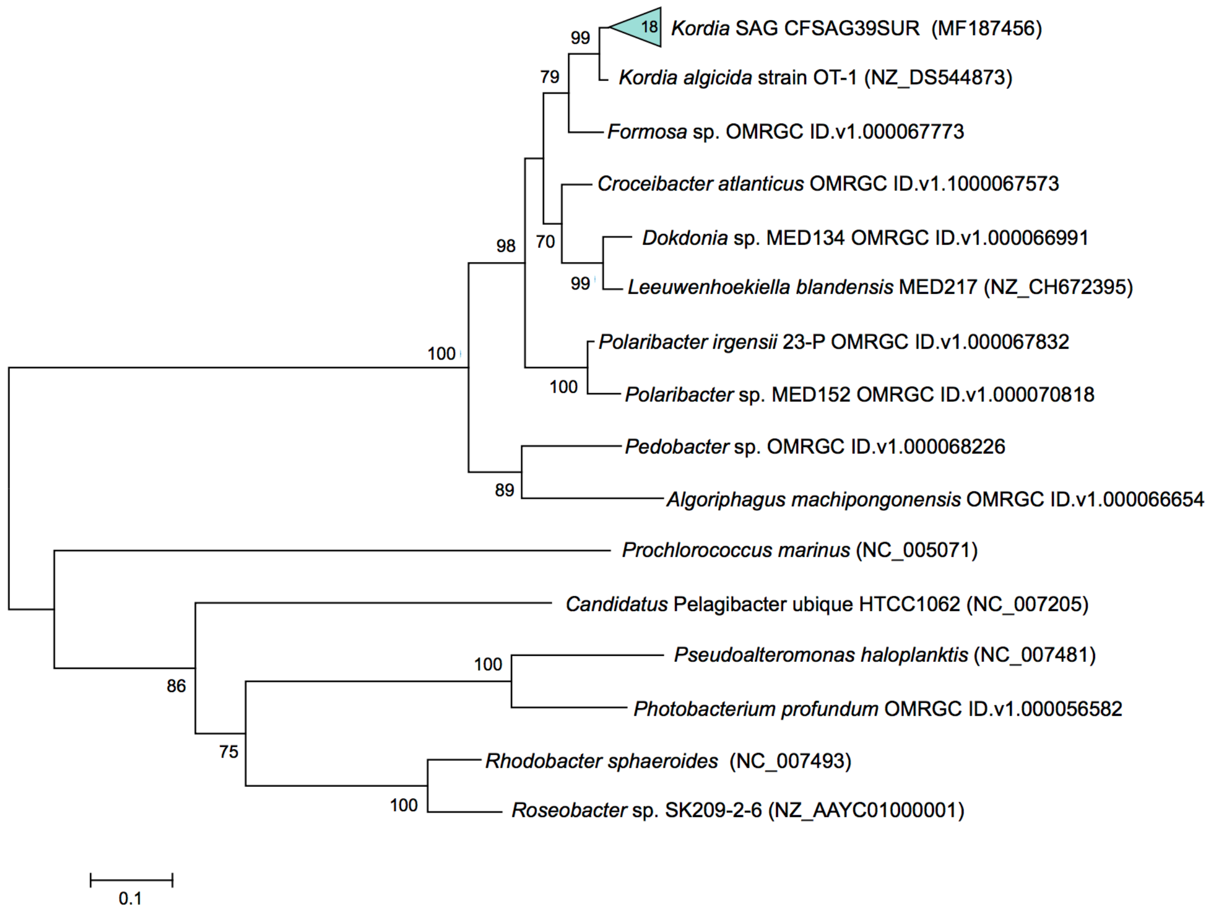

Figure 3. Maximum likelihood tree based on same length partial RpoB aminoacid sequences, showing relationships between the consensus sequence of the 18 identical *rpo*B genes from the *Kordia* SAGs, the closest hits from the Ocean Microbiome Reference Gene Catalog (OMRGC) (Sunagawa et al., 2015) and other sequences retrieved from the JGI IMG Database. Cyanobacteria and Proteobacteria act as outgroups and only bootstrap values ≥ 70% are shown at the nodes.

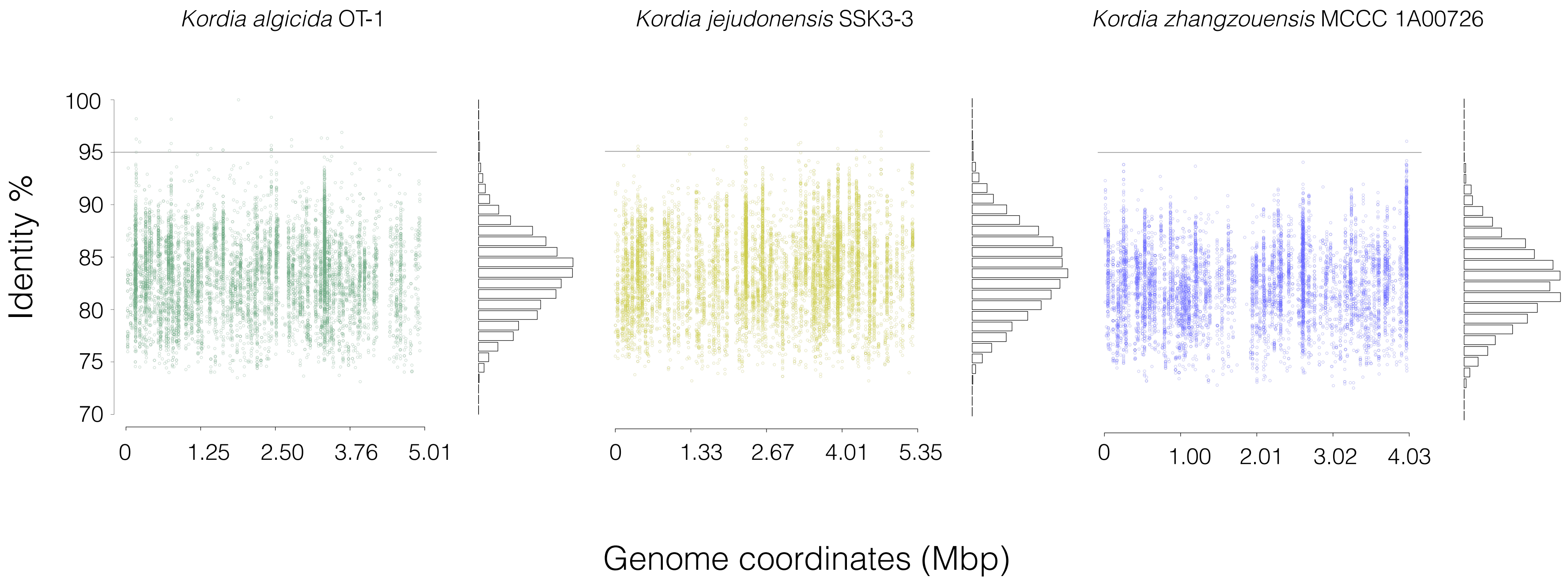

Figure 4. FRA plots for three of the four available *Kordia* genomes mapped against ST_085 mesopelagic sample (270 m, 0.2-3 μm). Metagenomic reads are displayed according to their position against de reference genome (X axis, genome coordinates) and their identity percentage (Y axis). The grey line located at 95% identity in the Y axis corresponds to 95% average nucleotide identity (ANI), threshold for bacterial species categorization. The bars on the right show the amount of reads mapped at each identity percentage (note different scales). These quantitative values are normalized to the sequencing depth and genomic size. Duplicates and reads mapped to the ribosomal operon have been removed to avoid bias. Plots for fourth *Kordia* genome, AAA285-F05 can be found at Figure 5 of this article.

Table 1. Samples used in this study.

Expedition	Sample ID	Station	Size Fraction (µm)	Depth (m)	Latitude	Longitude	Ocean	Kind of sample
Tara Oceans	39SUR	TARA_039	All	5	18.59	66.62	Indian	Single Cell Genomics
Tara Oceans	39DCM	TARA_039	0.2-1.6	25	18.57	66.48	Indian	Metagenomics
Tara Oceans	39MES	TARA_039	0.2-1.6	270	18.57	66.48	Indian	Metagenomics
Tara Oceans	39DCM	TARA_039	1.6-20	25	18.57	66.48	Indian	Metagenomics
Tara Oceans	85SUR	TARA_085	0.2-3	5	-62.03	-49.53	Southern	Metagenomics
Tara Oceans	85DCM	TARA_085	0.2-3	90	-62.03	-49.53	Southern	Metagenomics
Tara Oceans	85MES	TARA_085	0.2-3	790	-62.03	-49.53	Southern	Metagenomics
Malaspina	MP0326	20	0.8-20	4,000	-9.12	-30.19	Atlantic	Metagenomics
Malaspina	MP0327	20	0.2-0.8	4,000	-9.12	-30.19	Atlantic	Metagenomics
Malaspina	MP1493	82	0.8-20	2,150	-25.49	-179.52	Pacific	Metagenomics
Malaspina	MP1494	82	0.2-0.8	2,150	-25.49	-179.52	Pacific	Metagenomics

Table 2. Primers used in this study.

Primer name	Target gene	Sequence (5'-3')	Reference
27F	16S rRNA gene	5'-AGRGTTYGATYMTGGCTCAG-3'	Page et al., 2004
907R	16S rRNA gene	5'-CCGTCAATTCMTTTRAGTTT-3'	Lane et al., 1998
358F	16S rRNA gene	5'-CCTACGGGAGGCAGCAG-3'	Muyzer et al., 1993
CF434R	23S rRNA gene	5'-CACTATCGGTCTCTCAGG-3'	Acinas et al. 2014
rpoB-F	Beta Subunit of RNA polymerase (*rpo*B)	5'-ATTCCTTTYAARGGDTCDTGGAT-3'	This study
rpoB-R	Beta Subunit of RNA polymerase (*rpo*B)	5'-CCAATGTTTGGTCCTTCT-3'	This study
PR-Flavo-F	Proteorhodopsin	5'-GAYTAYGTWGSWTTYACDTTYTTTGTRGG-3'	Yoshizawa et al., 2012
PR-Flavo-R	Proteorhodopsin	5'-GCCCAWCCHACWARWACRAACCARCATA-3'	Yoshizawa et al., 2012

Table 3. Genome quality estimations of FRA reference genomes using *checkM* (Parks et al., 2015) and *fetchMG* (Sunagawa et al., 2013).

	Kordia AAA285-F05	*Kordia algicida*	*Kordia jejudonensis*	*Kordia zhangzhouensis*
Genome size (bp)	283,580	5,019,836	5,356,465	4,031,603
checkM				
Marker lineage	root_(UID1)	k__Bacteria_(UID203)	k__Bacteria_(UID203)	k__Bacteria_(UID203)
# genomes as reference	5656	5449	5449	5449
# reference markers	56	104	104	104
# markers found 0 times	56	0	0	0
# markers found 1 time	0	104	104	104
# markers found > 1 time	0	0	0	0
completeness %	0.00	100.00	100.00	100.00
contamination %	0.00	0.00	0.00	0.00
fetchMG				
# reference COGs	40	40	40	40
# COGs found 0 times	40	0	0	0
# COGs found 1 time	0	40	40	40
# COGs found > 1 time	0	0	0	0

Table 4. FRA values of ten different metagenomes from expeditions *Tara* Oceans and Malaspina 2010 mapped against 4 reference *Kordia* genomes.

***Kordia* AAA285-F05**

Metagenomic sample	Fraction (µm)	Depth (m)	Sequencing Depth	70-94.9% identity					95-100% identity				
				Number or recruited reads	read/ genome bp	recruitment per Mbp	normalized by subsampled Seq. Depth	% of recruited reads per Mbp	Number or recruited reads	read/ genome bp	recruitment per Mbp	normalized by subsampled Seq. Depth	% of recruited reads per Mbp
TARA_039_DCM	0.2-1.6	25	141787219	197	6,95E-04	6,95E+02	6,26E+01	4,90E-04	0	0,00E+00	0,00E+00	0,00E+00	0,00E+00
TARA_039_DCM	1.6-20	25	176193015	66	2,33E-04	2,33E+02	1,69E+01	1,32E-04	0	0,00E+00	0,00E+00	0,00E+00	0,00E+00
TARA_039_MES	0.2-1.6	270	83476538	13368	4,71E-02	4,71E+04	7,21E+03	5,65E-02	172	6,07E-04	6,07E+02	9,28E+01	7,27E-04
TARA_085_DCM	0.2-3	90	151217436	3327	1,17E-02	1,17E+04	9,91E+02	7,76E-03	12	4,23E-05	4,23E+01	3,57E+00	2,80E-05
TARA_085_MES	0.2-3	790	134267400	145859	5,14E-01	5,14E+05	4,89E+04	3,83E-01	23284	8,21E-02	8,21E+04	7,81E+03	6,12E-02
TARA_085_SUR	0.2-3	5	143758287	600	2,12E-03	2,12E+03	1,88E+02	1,47E-03	0	0,00E+00	0,00E+00	0,00E+00	0,00E+00
MP0326	0.8-20	4000	16469268	1890	6,66E-03	6,66E+03	5,17E+03	4,05E-02	2063	7,27E-03	7,27E+03	5,64E+03	4,42E-02
MP0327	0.2-0.8	4000	23070154	19818	6,99E-02	6,99E+04	3,87E+04	3,03E-01	33385	1,18E-01	1,18E+05	6,52E+04	5,10E-01
MP1493	0.8-20	4000	20947396	1020	3,60E-03	3,60E+03	2,19E+03	1,72E-02	1074	3,79E-03	3,79E+03	2,31E+03	1,81E-02
MP1494	0.2-0.8	4000	12772018	10068	3,55E-02	3,55E+04	3,55E+04	2,78E-01	16785	5,92E-02	5,92E+04	5,92E+04	4,63E-01

Kordia algicida

Metagenomic sample	Fraction (µm)	Depth (m)	Sequencing Depth	70-94.9% identity					95-100% identity				
				Number or recruited reads	read/ genome bp	recruitment per Mbp	normalized by subsampled Seq. Depth	% of recruited reads per Mbp	Number or recruited reads	read/ genome bp	recruitment per Mbp	normalized by subsampled Seq. Depth	% of recruited reads per Mbp
TARA_039_DCM	0.2-1.6	25	141787219	0	0,00E+00	0,00E+00	0,00E+00	0,00E+00	0	0,00E+00	0,00E+00	0,00E+00	0,00E+00
TARA_039_DCM	1.6-20	25	176193015	1179	2,35E-04	2,35E+02	1,70E+01	1,33E-04	6	1,20E-06	1,20E+00	8,66E-02	6,78E-07
TARA_039_MES	0.2-1.6	270	83476538	496	9,88E-05	9,88E+01	1,51E+01	1,18E-04	0	0,00E+00	0,00E+00	0,00E+00	0,00E+00
TARA_085_DCM	0.2-3	90	151217436	35259	7,02E-03	7,02E+03	5,93E+02	4,64E-03	15	2,99E-06	2,99E+00	2,52E-01	1,98E-06
TARA_085_MES	0.2-3	790	134267400	10183	2,03E-03	2,03E+03	1,93E+02	1,51E-03	23	4,58E-06	4,58E+00	4,36E-01	3,41E-06
TARA_085_SUR	0.2-3	5	143758287	54922	1,09E-02	1,09E+04	9,72E+02	7,61E-03	13	2,59E-06	2,59E+00	2,30E-01	1,80E-06
MP0326	0.8-20	4000	16469268	391	7,79E-05	7,79E+01	6,04E+01	4,73E-04	4	7,97E-07	7,97E-01	6,18E-01	4,84E-06
MP0327	0.2-0.8	4000	23070154	775	1,54E-04	1,54E+02	8,55E+01	6,69E-04	7	1,39E-06	1,39E+00	7,72E-01	6,04E-06
MP1493	0.8-20	4000	20947396	290	5,78E-05	5,78E+01	3,52E+01	2,76E-04	1	1,99E-07	1,99E-01	1,21E-01	9,51E-07
MP1494	0.2-0.8	4000	12772018	205	4,08E-05	4,08E+01	4,08E+01	3,20E-04	0	0,00E+00	0,00E+00	0,00E+00	0,00E+00

Kordia jejudonensis

Metagenomic sample	Fraction (µm)	Depth	Sequencing Depth	70-94.9% identity					95-100% identity				
				Number or recruited reads	read/ genome bp	recruitment per Mbp	normalized by subsampled Seq. Depth	% of recruited reads per Mbp	Number or recruited reads	read/ genome bp	recruitment per Mbp	normalized by subsampled Seq. Depth	% of recruited reads per Mbp
TARA_039_DCM	0.2-1.6	25	141787219	1852	3,46E-04	3,46E+02	3,11E+01	2,44E-04	2	3,73E-07	3,73E-01	3,36E-02	2,63E-07
TARA_039_DCM	1.6-20	25	176193015	1072	2,00E-04	2,00E+02	1,45E+01	1,14E-04	3	5,60E-07	5,60E-01	4,06E-02	3,18E-07
TARA_039_MES	0.2-1.6	270	83476538	773	1,44E-04	1,44E+02	2,21E+01	1,73E-04	4	7,47E-07	7,47E-01	1,14E-01	8,95E-07
TARA_085_DCM	0.2-3	90	151217436	40385	7,54E-03	7,54E+03	6,37E+02	4,99E-03	18	3,36E-06	3,36E+00	2,84E-01	2,22E-06
TARA_085_MES	0.2-3	790	134267400	11502	2,15E-03	2,15E+03	2,04E+02	1,60E-03	16	2,99E-06	2,99E+00	2,84E-01	2,22E-06
TARA_085_SUR	0.2-3	5	143758287	66201	1,24E-02	1,24E+04	1,10E+03	8,60E-03	19	3,55E-06	3,55E+00	3,15E-01	2,47E-06
MP0326	0.8-20	4000	16469268	408	7,62E-05	7,62E+01	5,91E+01	4,62E-04	1	1,87E-07	1,87E-01	1,45E-01	1,13E-06
MP0327	0.2-0.8	4000	23070154	841	1,57E-04	1,57E+02	8,69E+01	6,81E-04	5	9,33E-07	9,33E-01	5,17E-01	4,05E-06
MP1493	0.8-20	4000	20947396	297	5,54E-05	5,54E+01	3,38E+01	2,65E-04	0	0,00E+00	0,00E+00	0,00E+00	0,00E+00
MP1494	0.2-0.8	4000	12772018	235	4,39E-05	4,39E+01	4,39E+01	3,44E-04	2	3,73E-07	3,73E-01	3,73E-01	2,92E-06

Kordia zhangzhouensis

Metagenomic sample	Fraction (µm)	Depth	Sequencing Depth	70-94.9% identity					95-100% identity				
				Number or recruited reads	read/ genome bp	recruitment per Mbp	normalized by subsampled Seq. Depth	% of recruited reads per Mbp	Number or recruited reads	read/ genome bp	recruitment per Mbp	normalized by subsampled Seq. Depth	% of recruited reads per Mbp
TARA_039_DCM	0.2-1.6	25	141787219	2516	6,24E-04	6,24E+02	5,62E+01	4,40E-04	0	0,00E+00	0,00E+00	0,00E+00	0,00E+00
TARA_039_DCM	1.6-20	25	176193015	1275	3,16E-04	3,16E+02	2,29E+01	1,79E-04	0	0,00E+00	0,00E+00	0,00E+00	0,00E+00
TARA_039_MES	0.2-1.6	270	83476538	631	1,57E-04	1,57E+02	2,39E+01	1,87E-04	3	7,44E-07	7,44E-01	1,14E-01	8,91E-07
TARA_085_DCM	0.2-3	90	151217436	33545	8,32E-03	8,32E+03	7,03E+02	5,50E-03	0	0,00E+00	0,00E+00	0,00E+00	0,00E+00
TARA_085_MES	0.2-3	790	134267400	8445	2,09E-03	2,09E+03	1,99E+02	1,56E-03	1	2,48E-07	2,48E-01	2,36E-02	1,85E-07
TARA_085_SUR	0.2-3	5	143758287	50153	1,24E-02	1,24E+04	1,11E+03	8,65E-03	1	2,48E-07	2,48E-01	2,20E-02	1,73E-07
MP0326	0.8-20	4000	16469268	431	1,07E-04	1,07E+02	8,29E+01	6,49E-04	1	2,48E-07	2,48E-01	1,92E-01	1,51E-06
MP0327	0.2-0.8	4000	23070154	869	2,16E-04	2,16E+02	1,19E+02	9,34E-04	8	1,98E-06	1,98E+00	1,10E+00	8,60E-06
MP1493	0.8-20	4000	20947396	261	6,47E-05	6,47E+01	3,95E+01	3,09E-04	2	4,96E-07	4,96E-01	3,02E-01	2,37E-06
MP1494	0.2-0.8	4000	12772018	215	5,33E-05	5,33E+01	5,33E+01	4,18E-04	1	2,48E-07	2,48E-01	2,48E-01	1,94E-06

References

Acinas, S. G., Ferrera, I., Sarmento, H., Díez-Vives, C., Forn, I., Ruiz-González, C., et al. (2014). Validation of a new catalysed reporter deposition-fluorescence in situ hybridization probe for the accurate quantification of marine Bacteroidetes populations. *Environ. Microbiol.* doi:10.1111/1462-2920.12517

Lane, D.J., in *Nucleic acid techniques in bacterial systematics,* E. Stackebrandt, M. Goodfellow, Eds. (John Wiley & Sons, Chichester, UK, 1991), pp. 115–175.

Muyzer, G., Waal, E. C. D. E., and Uitierlinden, A. G. (1993). Profiling of Complex Microbial Populations by Denaturing Gradient Gel Electrophoresis Analysis of Polymerase Chain Reaction-Amplified Genes Coding for 16S rRNA. *Appl. Environ. Microbiol* 59, 695–700.

Page, K.A., Connon, S.A., Giovannoni, S.J. (2004), Representative freshwater bacterioplankton isolated from Crater Lake, Oregon. *Appl. Environ. Microbiol.* **70**, 6542–6550. doi:10.1128/AEM.70.11.6542-6550.2004

Parks, D.H., Imelfort, M., Skennerton, C.T., Hugenholtz, P., Tyson, G.W. (2015), CheckM: assessing the quality of microbial genomes recovered from isolates, single cells, and metagenomes. *Genome Research*. 25: 1043–1055.

Sunagawa, S., Mende, D.R., Zeller, G., Izquierdo-Carrasco, F., Berger, S.A., Kultima, J.R., Coelho, L.P., Arumugam, M., Tap, J., Nielsen, H.B., Rasmussen, S., Brunak, S., Pedersen, O., Guarner, F., de Vos, W.M., Wang, J., Li, J., Doré, J., Ehrlich, S.D., Stamatakis, A., Bork, P. (2013) Metagenomic species profiling using universal phylogenetic marker genes. *Nat. Meth.* 10, 1196–1199. doi:10.1038/nmeth.2693

Sunagawa, S., Coelho, L. P., Chaffron, S., Kultima, J. R., Labadie, K., Salazar, G., et al. (2015). Structure and function of the global ocean microbiome. *Science (80-.).* 348, 1261359–1261359. doi:10.1126/science.1261359.

Yoshizawa, S., Kawanabe, A., Ito, H., Kandori, H., and Kogure, K. (2012). Diversity and functional analysis of proteorhodopsin in marine Flavobacteria. *Environ. Microbiol.* 14, 1240–8. doi:10.1111/j.1462-2920.2012.02702.x.
